# Horizontal Transmission of *Candida albicans* and Evidence of a Vaccine Response in Mice Colonized with the Fungus

**DOI:** 10.1371/journal.pone.0022030

**Published:** 2011-07-19

**Authors:** Jim E. Cutler, Miriam Corti, Patrick Lambert, Michael Ferris, Hong Xin

**Affiliations:** 1 Department of Pediatrics, Louisiana State University Health Sciences Center, New Orleans, Louisiana, United States of America; 2 Research Institute for Children at Children's Hospital, New Orleans, Louisiana, United States of America; Albert Einstein College of Medicine, United States of America

## Abstract

Disseminated candidiasis is the third leading nosocomial blood stream infection in the United States and is often fatal. We previously showed that disseminated candidiasis was preventable in normal mice by immunization with either a glycopeptide or a peptide synthetic vaccine, both of which were *Candida albicans* cell wall derived. A weakness of these studies is that, unlike humans, mice do not have a *C. albicans* GI flora and they lack *Candida* serum antibodies. We examined the influence of *C. albicans* GI tract colonization and serum antibodies on mouse vaccination responses to the peptide, Fba, derived from fructose bisphosphate aldolase which has cytosolic and cell wall distributions in the fungus. We evaluated the effect of live *C. albicans* in drinking water and antimicrobial agents on establishment of *Candida* colonization of the mouse GI tract. Body mass, *C. albicans* in feces, and fungal-specific serum antibodies were monitored longitudinally. Unexpectedly, *C. albicans* colonization occurred in mice that received only antibiotics in their drinking water, *provided that* the mice were housed in the same room as intentionally colonized mice. The fungal strain in unintentionally colonized mice appeared identical to the strain used for intentional GI-tract colonization. This is the first report of horizontal transmission and spontaneous *C. albicans* colonization in mice. Importantly, many *Candida*-colonized mice developed serum fungal-specific antibodies. Despite the GI-tract colonization and presence of serum antibodies, the animals made antibodies in response to the Fba immunogen. This mouse model has potential for elucidating *C. albicans* horizontal transmission and for exploring factors that induce host defense against disseminated candidiasis. Furthermore, a combined protracted GI-tract colonization with *Candida* and the possibility of serum antibody responses to the presence of the fungus makes this an attractive mouse model for testing the efficacy of vaccines designed to prevent human disseminated candidiasis.

## Introduction

Disseminated candidiasis is the third leading nosocomial blood stream infection in the United States [1]. Despite availability of several drugs that are inhibitory to *Candida* spp., which cause disseminated candidiasis, over 40% of treated patients die of this disease [2], [3]. Development of an anti-candidiasis vaccine offers a preventive approach to reducing the incidence of disease [4], [5]. Several groups have been working toward an anti-*Candida* vaccine. Some have focused on cell mediated immunity as the mechanism of protection [6], which is in accordance with the literature [7]–[8], whereas we [4], [9] and others [5], [10], [11] have determined that antibodies specific to certain *Candida* antigens are protective.

The antibody-mediated protection approach against candidiasis has been controversial because candidiasis patients and experimental animals with *Candida-*specific antibodies may not show evidence of disease resistance. Furthermore, intact cells of *C. albicans,* the prevalent specific cause of disseminated candidiasis, may or may not induce protective antibody responses in experimental animals [12]. We've shown that specific anti-mannan antibodies are protective if they recognize β-1,2-mannotriose or –mannobiose moieties of the phosphomannoprotein complex, whereas antibodies specific for α-linked mannan of the same complex are not protective [13]. The important point, from the standpoint of antibody-mediated protection, is that the enormous antigenic complexity of the *Candida* cell [14]–[18] should not be expected to reliably induce production of protective antibodies, as defined by specificity, titer, isotype and effector function.

Rather than providing the host with a highly complex array of *Candida* antigens, a more predictive protective response should be inducible by a vaccine comprised of a limited number of defined specific antigens. To that end, we developed a completely synthetic vaccine consisting of a β-1,2-mannotriose linked via a non-immunogenic tether to a peptide derived from a protein associated with cytosolic and cell wall compartments of *C. albicans* [4]. Antibodies induced to the beta-linked mannotriose equally recognize both beta-mannotriose and beta-mannobiose [19] and are protective against disseminated candidiasis. These beta-oligomannosides are produced by *C. albicans* and several additional *Candida* species [9], [20]–[23]. The peptide chosen for the current study was designated as Fba and is a 14 amino acid sequence located in the *N-*terminal portion of fructose bisphosphoaldolase. This peptide was originally tested for its' putative T-cell carrier effect in facilitation of induction of antibody against the beta-mannotriose. Surprisingly, mice immunized against the mannotriose-Fba glycoconjugate made antibody against both moieties [4] and animals immunized against the Fba alone also produced antibodies against the peptide. Here we employ the peptide alone as the *Candida-*specific immunogen, which, as explained below, allowed us to distinguish host response to *Candida* colonization of the GI-tract from immunogen-induced antibody responses.

Our heretofore use of normal mice in protection studies has two important limitations. First, humans are often colonized with *C. albicans* in the GI-tract, while other animals [24] including normal laboratory mice are not. Second, most humans have a complex array of polyclonal *C. albicans-*specific antibodies circulating in their blood-vascular system, whereas normal mice do not. These limitations have raised questions as to whether GI tract colonization with the fungus and/or antibody responses to prior or present colonization will mitigate a vaccine response [12].

Adult mouse GI-tract colonization with *C. albicans* sustained for 2–3 weeks has been achieved by a number of investigators [25]–[31]. In an attempt to study the effects of *C. albicans* colonization over several months, we began by following a recently described protocol [32], and incorporated several modifications. The modified protocol resulted in a prolonged colonization that lasted over 80 days. The colonization can lead to the appearance of serum antibodies specific for *C. albicans*. Surprisingly, mice treated with oral antibacterial agents were found to be susceptible to horizontal transmission and GI-tract colonization of the fungus transmitted from intentionally colonized animals. Furthermore, we showed that the *Candida-*colonized mice were still capable of producing antibodies in response to a peptide vaccine against candidiasis. For the first time, novel vaccine formulations and delivery systems acceptable for human use may be tested in mice that have a protracted *C. albicans* colonization of the GI tract and anti-fungal antibodies in their blood. In addition, this animal model may be useful for studies on host-to-host transmission of the fungus.

## Results

### Pilot experiment: Evidence for protracted colonization and antibody production

**Figure 1:**
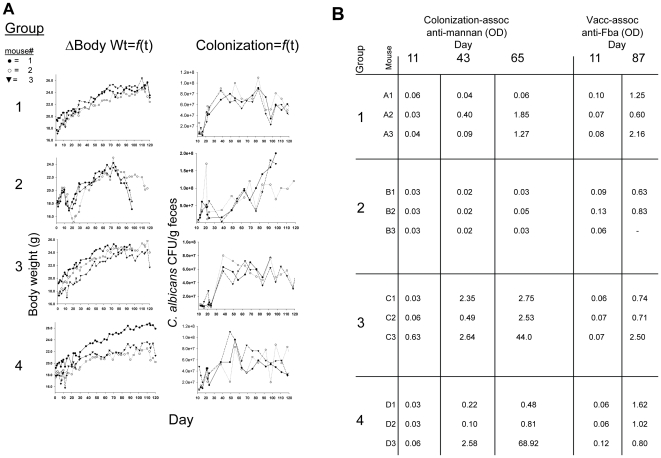
Body mass, GI-tract colonization and appearance of *Candida albicans-*specific antibodies as a function of time. Mice were given antibiotics in their water prior to and during ingestion of live *C.albicans.* Changes in body weight were monitored throughout the experiment, as was *C. albicans* colonization as measured by fungal colony forming units (CFU) per gram weight of feces (Panel A). One group was immunosuppressed (group 2) and all were followed for serum anti-mannan antibody induced as a result of GI-tract colonization. All mice were immunized and appearance of serum antibody against the vaccine immunogen, Fba, indicated whether they responded to the vaccine (Panel B, compare day 11 before vaccination to day 87 after vaccination). Groups 1 and 2 received antibacterial agents and fluconazole, but only group 2 was treated with cyclophosphamide. Group 3 was similar to 1, except that 3 did not receive fluconazole. Group 4 was not pretreated with antimicrobial agents prior to ingesting *C. albicans,* and this group was not maintained on gentamicin. Antibody responses were measured by ELISA for a 1∶100 dilution of each serum and are expressed as OD_490nm._ Shown are antibody responses at days 11, 43 and 65 to a mannan fraction (α-mannan), which occurred as a result of GI-tract colonization, and antibody against Fba peptide, which occurred as a result of Fba vaccination (Panel B). In this experiment all mice were vaccinated on day 51 and boosted on days 65 and 79.

In a pilot experiment of four groups of three C57BL/6 mice each, the variables were fluconazole, cyclophosphamide, no antibiotics prior to *C. albicans* ingestion, and gentamicin (Table 1). These variables were selected as based on the mouse model previously described [32]. Groups 1 and 2 were treated with antibacterial and antifungal agents prior to five days of oral ingestion of live *C. albicans.* In addition, group 2 received an immunosuppressive drug after the five day ingestion period. Group 3 received only antibacterial agents and group 4 did not receive antibiotics prior to the five day period of ingesting *C. albicans* (Table 1). All mice were immunized with the Fba peptide vaccine starting on day 51. Mice treated with antimicrobial agents prior to fungal ingestion appeared stressed by day 2 of the experiment as indicated by ruffled fur and weight-loss by some of the mice, but by around day 11 all of the animals except those that received the cyclophosphamide appeared healthy with smooth coat and weight increase (Fig. 1). Fecal samples obtained before and at day 4 of antibiotic treatment were homogenized in DPBS, plated onto trypticase soy agar containing 5% sheep blood and incubated aerobically at 37°C for up to 4 days. Homogenized feces from before and during treatment yielded heavy and no bacterial growth, respectively. Day 0 (before initiation of antibiotic therapy) and day 4 homogenized feces yielded no evidence of yeast colonies, whether or not they were treated with fluconazole, when plated onto glucose-yeast extract-peptone agar containing chloramphenicol. Following ingestion of *C. albicans* during days 5–9 of the experiment, *Candida* colonization was maintained throughout the remainder of the experiment in all groups, but was highest in group 2 animals that were treated three times with cyclophosphamide every other day starting on day 11. In a previous experiment by others [32], animals treated similarly to the group 2 animals in our experiment died by day 7 after cyclophosphamide immunosuppression. In our experiment, two mice in group 2 survived 100 days before becoming moribund and were sacrificed. We do not know the reason for the protracted survival of immunosuppressed mice in our experiment, except that in the Koh et al study, C3H/HeN mice were used as compared to C57BL/6 mice in our study [32].

**Table 1:** Pilot experiment treatment schedule examining multiple variables.

Water contents/Day									
	1	2	3	4	5–9	10+	11	13	15
Grp									
1	p/s/f	p/s/f	p/s/f	p/s/-	p/s/Ca	p/s/g			
2	p/s/f	p/s/f	p/s/f	p/s/-	p/s/Ca	p/s/g	cyclo	cyclo	cyclo
3	p/s/-	p/s/-	p/s/-	p/s/-	p/s/Ca	p/s/g			
4	-/-/-	-/-/-	-/-/-	-/-/-	p/s/Ca	p/s/-			

p, penicillin; s, streptomycin; f, fluconazole; Ca, *C. albicans;* g, gentamicin; cyclo, cyclophosphamide.

By day 43 of the experiment, which was 34 days after cessation of *C. albicans* in the water, serum antibodies specific for a 2-mercaptoethanol extract of *C. albicans* enriched for cell surface phosphomannoprotein complexes [33] were detected in one animal in group 1 and all animals in groups 3 and 4, but none in group 2 animals that received the cyclophosphamide immunosuppression treatments (Fig. 1, panel B). Groups 3 and 4 maintained the highest titers as indicated by absorbance of ELISA readings on a 1∶100 dilution of each serum sample. All mice were vaccinated with the Fba peptide on day 51 and serum antibodies specific for the Fba were detectable in all mice by day 87, with the cyclophosphamide-treated animals (group 2) giving the weakest response as expected (Fig. 1). Since the Fba vaccine induces antibodies that do not recognize epitopes in the mannan extract (data not shown), the increase of anti-mannan antibodies at day 65 was presumably due to the continued presence of *C. albicans* GI tract colonization. Likewise, as we show below, non-immunized mice colonized with the fungus do not develop anti-Fba antibodies, therefore the anti-Fba response was due to the vaccine.

This pilot experiment addressed a large number of variables, some of which were not adequately controlled. Nonetheless, the results provided at least preliminary indications that a protracted *C. albicans* colonization of the adult mouse GI tract is possible, colonization may lead to development of serum antibodies that recognize cell surface epitopes of the fungus and colonized antibody-producing mice may respond to the Fba vaccine by making antibodies specific to the Fba peptide.

### First follow-up experiment: Initial suggestion of horizontal transmission of *C. albicans*

**Figure 2:**
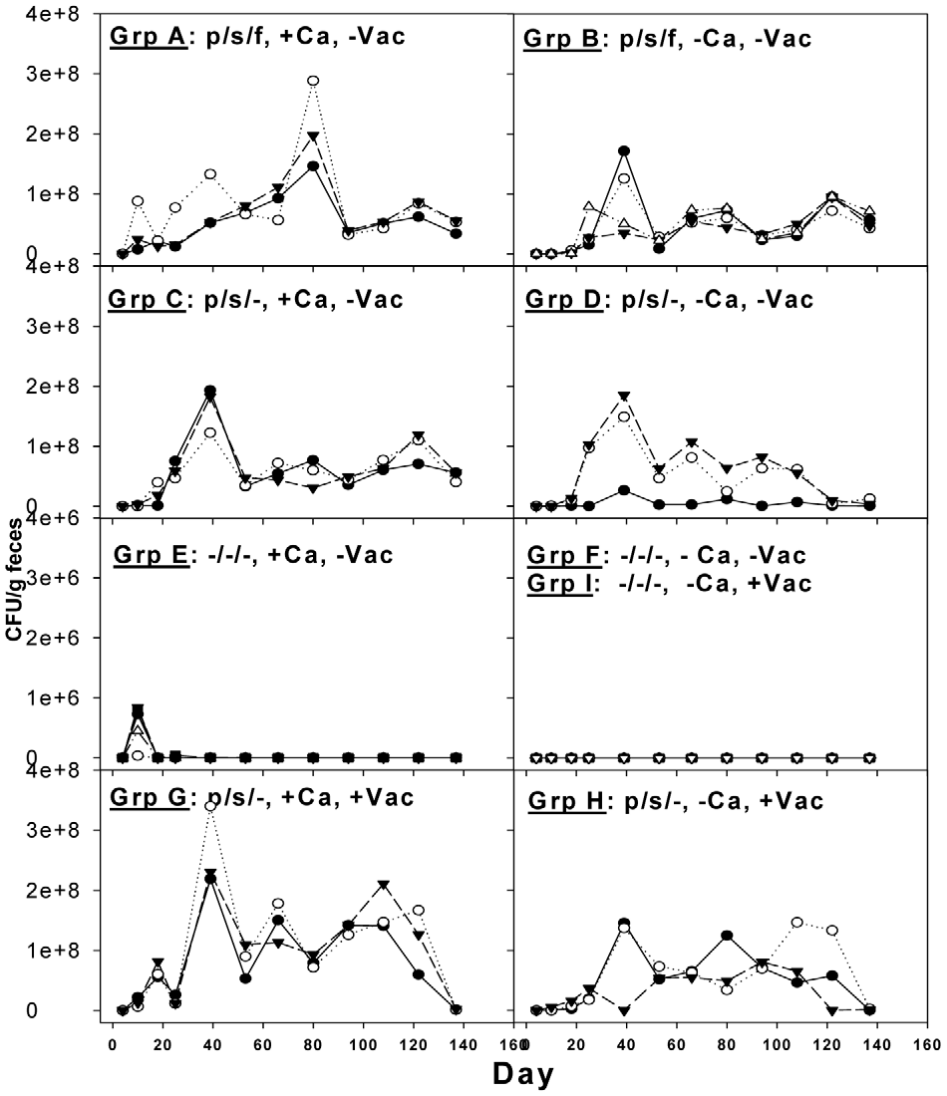
Antibacterial therapy is required for a protracted GI-tract colonization with *C. albicans*. Mice received oral administration of various combinations of antimicrobial agents, live *C. albicans,* and a peptide (Fba) vaccine. Control groups did not receive one or more of the treatments. All groups that received at least two of the antibacterial agents and were fed *C. albicans* for a five day period (e.g., groups A, C and G) developed a protracted GI-tract colonization with the fungus as evidenced by the presence of the fungus in fecal specimens. Antibiotics were required for the fungal colonization as shown by the presence of *C. albicans* in the feces of group E only during the five day feeding period. Surprisingly, antibiotic-treated mice that were not fed *C. albicans* also became colonized (groups B, D and H). These results suggested the possibility that antibiotic-treated mice are susceptible to horizontal transmission of the fungus. p = penicillin; s = streptomycin; f = fluconazole; +Ca = *C. albicans* in drinking water on days 5–9; +Vac = vaccinated with Fba. Each mouse in each group is represented by an open circle, closed circle or closed triangle.

The next experiment was performed using BALB/c mice and addressed the above variables, except that cyclophosphamide immunosuppression was not pursued. This mouse strain was chosen because of its' extensive use in our previous studies and to show that mice with different MHC haplotypes behave similarly. This experiment included more extensive controls, such as presence or absence of *C. albicans* in the drinking water (Table 2). As with the C57BL/6 mice in the pilot experiment described above, plating of undiluted homogenized feces yielded confluent growth of bacteria prior to the start of antibiotics, but no growth by day 4 of antibiotic treatment. Likewise, no evidence of viable yeast was found in feces prior to initiation of antibiotic treatment. As in the pilot experiment, *C. albicans* colonized the GI tract of BALB/c mice that were treated with antibiotics and received the fungus in their drinking water on days 5–9 (Fig. 2, groups A, C and G). Control mice that were not treated with antibiotics did not develop a sustained *Candida* flora following ingestion of the fungus (Fig. 2, group E). Unexpectedly, another control group that was treated with antibiotics, but not fed the fungus, developed a protracted fungal colonization (Fig. 2, groups B, D and H). This result was surprising because (normal) mice not treated with antibiotic water were highly resistant to prolonged GI-tract colonization even when *C. albicans* was added to the drinking water (Fig. 2, group E). Indeed, this is the first observation on an apparent spontaneous colonization of the mouse GI tract with *C. albicans.* Furthermore, the antibiotic dependency of this result was confirmed by groups F and I (Fig.2) that did not receive antibiotics in their water, and they did not have fungi in their stool. This experiment was thus confounded by spontaneous development of GI-tract colonization in groups B, D and H and raised the question as to the identity and source of the colonizing fungus in these groups.

**Table 2:** Experiment leading to preliminary indications of horizontal transmission of *Candida albicans.*

		Water contents/Day					
		1	2	3	4	5–9	10+
Grp	Fba vac						
A	−	p/s/f	p/s/f	p/s/f	p/s/-	p/s/Ca	p/s/g
B	−	p/s/f	p/s/f	p/s/f	p/s/-	p/s/-	p/s/g
C	−	p/s/-	p/s/-	p/s/-	p/s/-	p/s/Ca	p/s/g
D	−	p/s/-	p/s/-	p/s/-	p/s/-	p/s/-	p/s/g
E	−	-/-/-	-/-/-	-/-/-	-/-/-	-/-/Ca	-/-/-
F”normal”	−	-/-/-	-/-/-	-/-/-	-/-/-	-/-/-	-/-/-
G	+	p/s/-	p/s/-	p/s/-	p/s/-	p/s/Ca	p/s/g
H	+	p/s/-	p/s/-	p/s/-	p/s/-	p/s/-	p/s/g
I”normal”	+	-/-/-	-/-/-	-/-/-	-/-/-	-/-/-	-/-/-

F and I”normal” represent the negative and positive normal mouse controls, respectively.

Growth on ChromAgar gave presumptive evidence that all randomly selected colonies from stool samples obtained from groups B, D and H, were *C. albicans.* All colonies produced the same green pigmentation typical of this species growing on this medium and the pigmentation was identical to that of the *C. albicans* strain SC5314 that was fed to the mice (data not shown). Essentially identical sequences were observed for all six PCR-amplified multilocus sequence typing (MLST) products of all randomly selected *C. albicans* isolates selected from mice that were either spontaneously or intentionally colonized. This strongly indicated that spontaneous colonization was due to the *C. albicans* strain used for intentional colonization. These results also suggested that mice that were not intentionally colonized (group B, D and H, Fig. 2) acquired *C. albicans* from intentionally-colonized mice that were housed in the same room. Throughout the experiment, all mice were kept exclusively in our AAALAC-certified animal facility complete with HEPA-filtered controlled air flow. In accordance with policy, each cage contained no more than five animals. All food, water, water bottles, cages and bedding were pre-sterilized, each cage was fitted with micro-barrier tops and the cages were kept in a full ventilation environmental housing system that provided HEPA filtered air supply and HEPA filtered exhaust (ACE Micro-Vent Environmental Systems, Allentown Caging Equipment Co, Allentown, NJ). In addition, all personnel wore PPE equipment including gloves, sterile gowns, masks, bonnets and shoe-booties. These approaches made cross contamination unlikely during undisturbed housing of the animals, but cross contamination was certainly a possibility during cage cleaning procedures and mouse handling during experimental procedures. To address the cross-contamination possibility, the experiment was set-up again, but this time we included housing a few duplicate groups in an isolated room as described below.

### Second follow-up experiment: Solid evidence for horizontal transmission

To address the *Candida* source question, the experiment on BALB/c mice was again set-up as shown in Table 2, but with duplication of groups B, D and H; the duplicates were housed in an isolated room in the vivarium and referred to as B', D' and H' (Tables 2 and 3). All other groups were housed in a common room as in the previous experiment. The mice were provided with specialized water (Tables 2 and 3), with B', D' and H' in isolation receiving identical water treatments as in common room groups B, D and H, respectively. Groups G, H, and H' were vaccinated with Fba-dendritic cells intraperitoneally starting with the priming dose on day 31 of the experiment, boosted with the same 14 days later and boosted 14 days later subcutaneously with Fba prepared as an emulsion in CFA. All non-vaccinated mice were given adjuvant alone subcutaneously as a control at the time of the second booster for the vaccinated groups (day 59). As in the previous experiment, facultative anaerobic bacterial growth in fecal specimens was inhibited by antibiotic treatment and there was no evidence of yeast in the feces of any mice prior to the start of antibiotic therapy. The animals were followed for weight changes, *Candida* CFU's in stool, serum antibodies specific for cell wall phosphomannoproteins and for Fba. This part of the experiment was terminated on day 80.

**Table 3:** Treatment schedule and results of experiment to address horizontal transmission and antibody responses due to GI-tract colonization and Fba immunization.

Grp	Fba vac	Water//+ or − Ca	Day 66	Day 71	Day 71
			Avg(SE) Ca/g feces	Avg(SE) anti-man	Avg(SE) anti-Fba
A	−	p/s/f//+Ca	1.76×10^7^(1.8×10^5^)	0.37(0.15)	0.16(0.02)
B	−	p/s/f//-Ca	2.98×10^7^(4.76×10^5^)	0.08(0.01)	0.20(0.03)
B'	−	p/s/f//-Ca	None detected	0.07(0.01)	0.19(0.02)
C	−	p/s/-//+Ca	3.41×10^7^(5.33×10^5^)	0.28(0.10)	0.19(0.01)
D	−	p/s/-//-Ca	5.44×10^7^(6.62×10^6^)	0.11(0.03)	0.22(0.01)
D'	−	p/s/-//-Ca	None detected	0.08(0.01)	0.23(0.02)
E	−	-/-/-//+Ca	4.02×10^4^(2.79×10^4^)	0.08(0.01)	0.25(0.01)
F”normal”	−	-/-/-//-Ca	None detected	0.08(0.01)	0.21(0.02)
G	+	p/s/-//+Ca	4.21×10^7^(4.58×10^6^)	0.44(0.11)	0.69(0.03)
H	+	p/s/-//-Ca	4.64×10^7^(4.58×10^6^)	0.13(0.04)	0.83(0.04)
H'	+	p/s/-//-Ca	None detected	0.12(0.03)	0.86(0.13)
I”normal”	+	-/-/-//-Ca	None detected	0.09(0.01)	0.83(0.02)

Grp designations are similar to Table 2, except B', D' and H' were in an isolated room. Immunization with Fba vaccine commenced on day 31, first booster day 45 and second booster day 59. All non-vac animals received adjuvant only on day 59.

**Figure 3:**
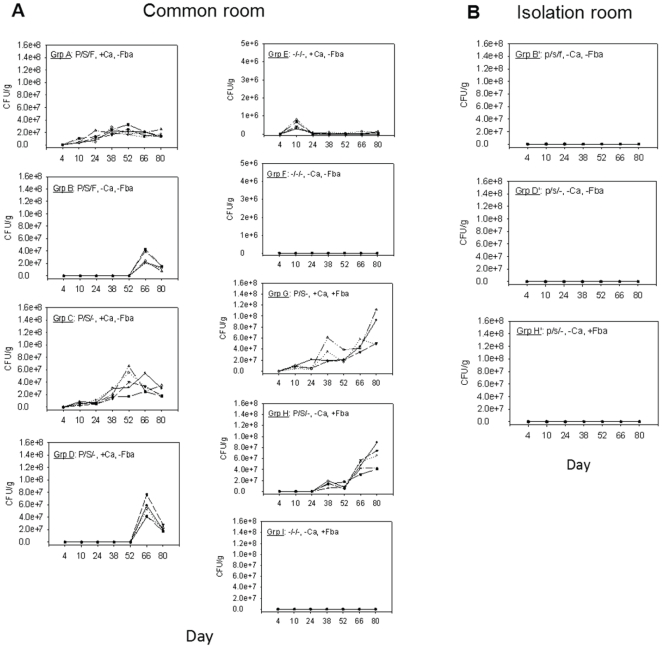
Confirmation of horizontal transmission of *C. albicans* in mice treated with antibacterial agents. Antimicrobial-treated mice not fed *C. albicans* (groups B, D and H) housed in a common room (Panel A) with other groups of mice intentionally colonized with the fungus (+Ca), began showing evidence of fungal colonization by day 38 (group H) or after day 52 (groups B and D). Duplicate groups of B, D and H kept in an isolated room (i.e., groups B', D' and H', respectively) did not show evidence of the fungus in their feces (Panel B). p = penicillin; s = streptomycin; f = fluconazole; +Ca = *C. albicans* in drinking water on days 5–9; +Fba = vaccinated with Fba starting on day 31. The various symbols on the curves represent each animal subject.

Antibiotic-treated mice showed an initial weight drop during the first few days as in the previous experiments, but then steadily gained weight until day 80. As in the previous experiment, regardless of whether they were fed *C. albicans* in the drinking water, all antibiotic-treated mice in the common room developed a fungal colonization of the GI tract by day 66 of the experiment (Fig. 3, panel A: groups A, B, C, D, G, and H).

More rigorous measures were taken in this experiment as compared to the previous experiment to reduce cross-contamination between the groups during handling of the mice. For example, personnel who handled the mice in the isolated room did so prior to entering the common room. In both rooms gloves were changed between cages, separate weighing vessels were used for each cage to determine mouse weights and to collect stool specimens. The stricter precautions in this experiment may have accounted for the delayed onset of *Candida* colonization of non-fungal fed animals that received antibiotics (compare Figs 2 and 3 for groups B, D and H), but as in the previous experiment colonization of non-*Candida* fed antibiotic-treated mice in the common room could not be prevented.

As in the previous experiment, colonies in stool samples from groups B, D and H were tested for appearance of growth on ChromAgar and for strain identity by multilocus sequencing. Again, ChromAgar growth was green and identical to that of the SC5314 that was used for intentional colonization. Multilocus sequencing was extended to include additional primers for strain comparisons [34], but the results of over 20 randomly-selected colonies were essentially identical with sequences obtained with the primers against the SC5314 strain.

The *Candida* colonization occurring in antibiotic-treated non-*Candida* fed mice kept in the common room, lack of colonization in isolated antibiotic-treated mice and colonization caused by the same strain in non-*Candida* fed mice as that in animals intentionally colonized provide evidence that antibiotic-treated mice are exquisitely susceptible to host-to-host transmission of the fungus. The mechanism of host-to-host transmission likely includes fomites contaminated with *C. albicans* intentionally colonized mice. Gloves, bedding aerosols, mouse restrainers used during bleedings and cross-cage fecal contamination during cage cleaning may well have played a role in the transmission of the fungus from intentionally colonized mice to antibiotic treated animals.

**Figure 4:**
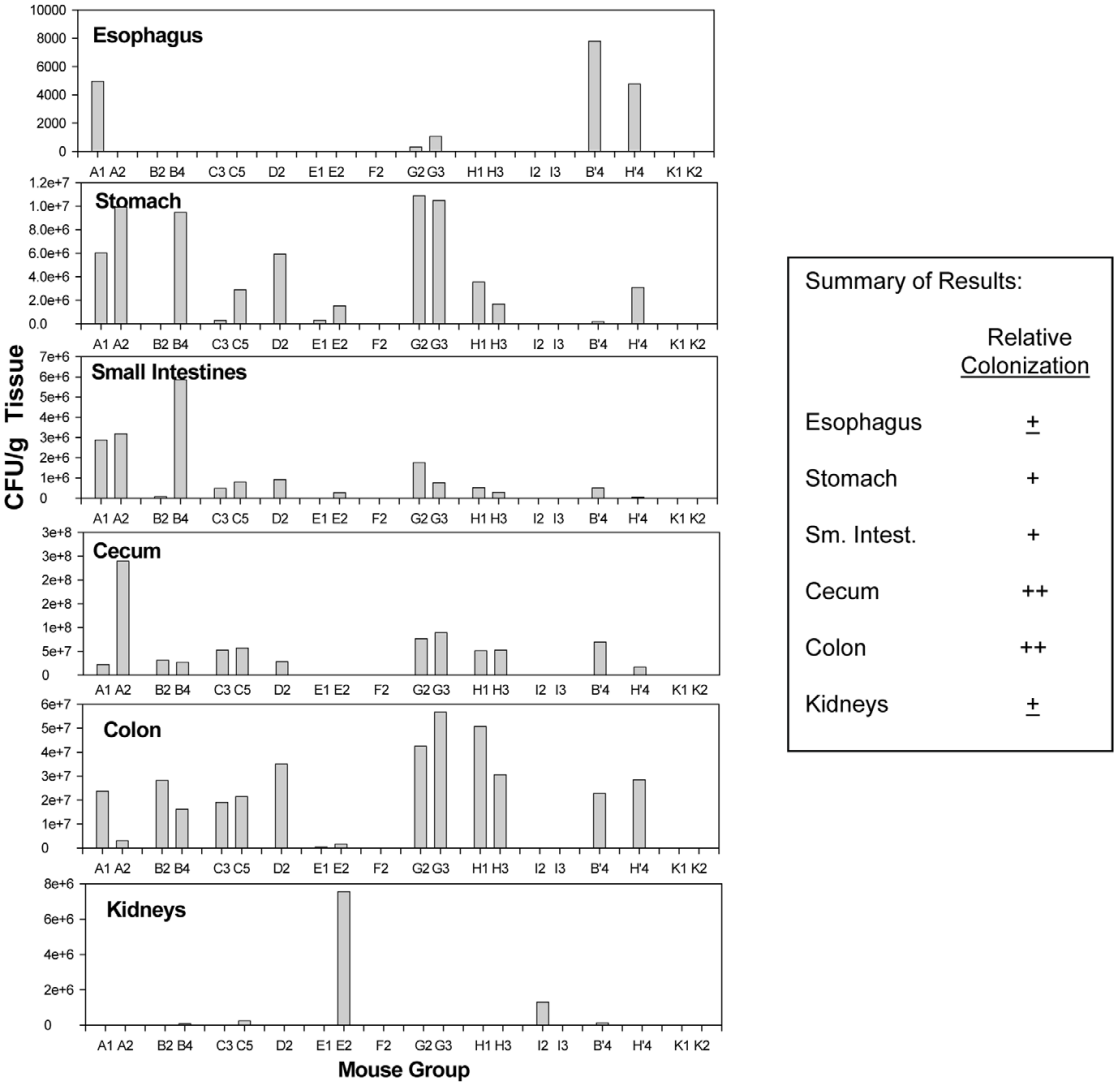
GI-tract colonization was especially profound in the cecum and large intestine. Mice that survived the experiment (Fig. 3), were sacrificed by CO_2_ asphyxiation, the GI-tract was sectioned into major components, homogenized and plated for fungal burden (*C. albicans* CFU/gram tissue). Each mouse per group that survived for processing are noted on the X-axis.

Mice that survived the experiment were sacrificed and analyzed for sites of colonization within the GI tract. Colonization on a per gram basis was light to non-existent in the esophagus, slightly more abundant in the stomach and small intestines, but especially high fungal presence was associated with the cecum and the large intestine (Fig. 4). These results are similar to those described by others [27] in short-term experiments on an outbred strain of adult mice.

**Figure 5:**
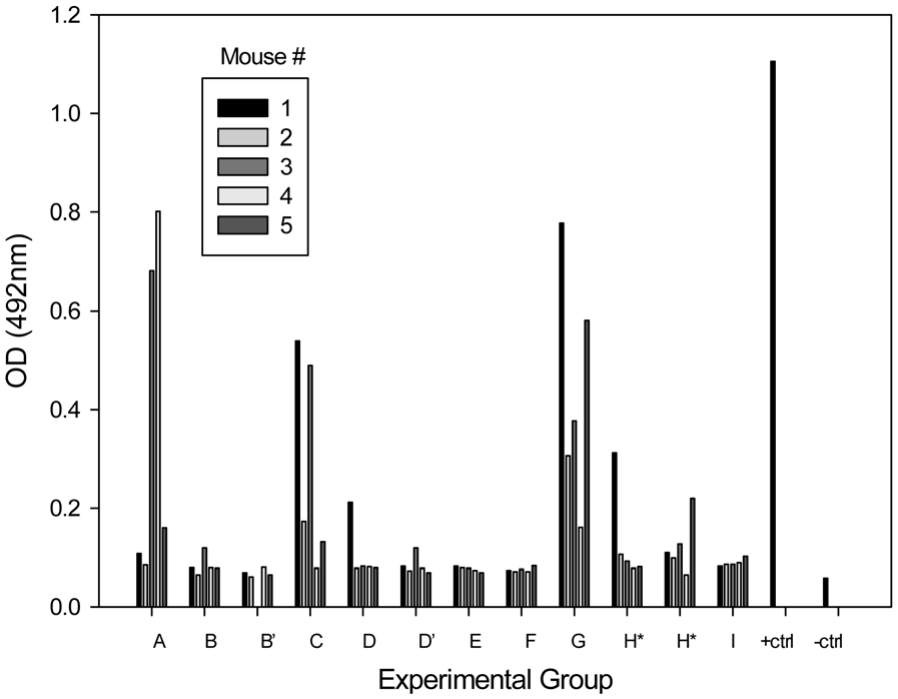
*C. albicans c*olonization of mice may result in appearance of serum antibodies specific for a mannan extract of the fungus. By day 71, serum antibody was especially obvious in mice that were treated with antimicrobial agents and received *C. albicans* in their drinking water (i.e., groups A, C and G;see Table 3). Only one mouse from each of two groups that became colonized later as a result of horizontal transmission had evidence of serum antibody (groups B and D). Note that the sera from groups H and H' were not properly marked for identification of H or H' and were, thus, both designated as H* and H*. The positive control (+ctrl) for the ELISA was monoclonal antibody B6.1, which is specific for a mannan component in the *C. albicans* cell wall. The negative control (-ctrl) contained all of the ELISA reagents except for primary antibody, which was omitted.

As determined in the pilot experiment, prior to immunization, colonized mice may show the appearance of antibodies specific for cell phosphomannoprotein complexes in their sera, but not to the vaccine peptide Fba. In this experiment, anti-phosphomannoprotein responses were detected in some of the colonized mice by day 71 of the experiment (Fig. 5). The most consistent responses (i.e., 5/5 mice) occurred in group G animals that received antibiotics and were intentionally colonized. Mice in groups A and C were next in titer and consistency. Only 3 of 15 mice in the antibiotic-treated animals that became spontaneously colonized (groups B, D and H) showed the presence of serum antibodies, but this might be due to the delay in colonization until around 60+ days as compared to colonization occurring during days 5–9 in mice that received the fungus in their water. The rules governing whether a colonized animal will develop serum antibodies are not obvious and require further investigation.

**Figure 6:**
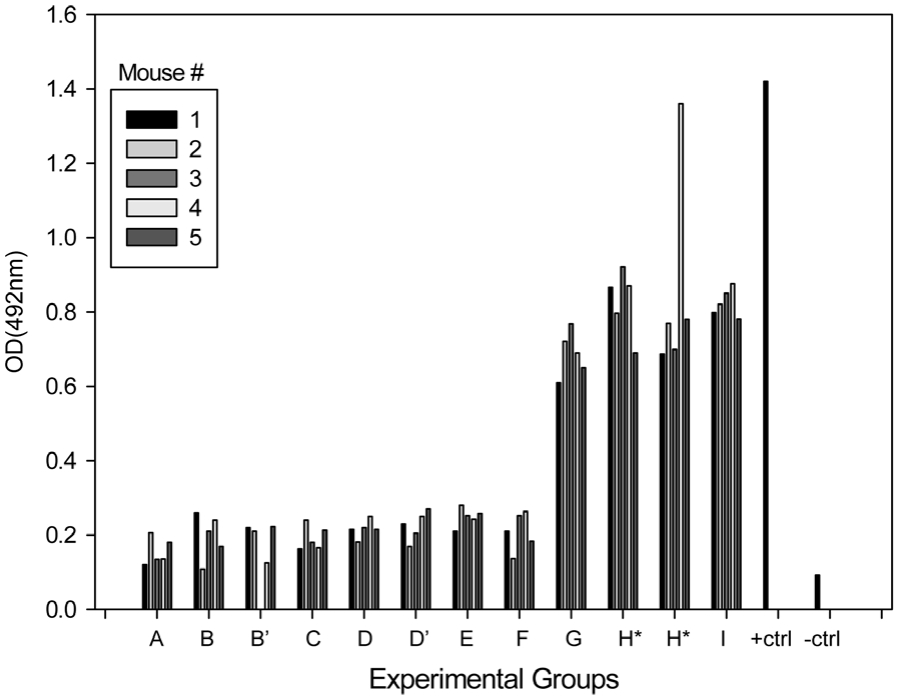
Vaccination induced appearance of serum antibodies regardless of colonization status. All mice immunized with the Fba vaccine (groups G, H, H' and I) developed serum antibodies against the Fba immunogen. Note that the sera from groups H and H' were not properly marked for identification of H or H' and were, thus, both designated as H* and H*.

In mice that received the vaccine starting on day 31 and ending with the second booster on day 59 (i.e., groups G, H, H' and I), antibody specific for the Fba peptide was detectable in their sera by day 71 (Fig. 6). Anti-Fba responses occurred in all animals in group G even though all mice in that group were also making antibody reactive with the phosphomannoprotein complexes from the cell wall as a result of colonization (Fig. 5).

These data indicate that the Fba vaccine can induce specific anti-vaccine responses in animals colonized with *C. albicans* in their GI tract and have circulating antibodies specific for the fungus prior to immunization.

## Discussion

### Characterization of the colonization model

Three independent experiments showed that a protracted *C. albicans* GI-tract colonization in adult inbred strains of mice could be reliably induced. The colonization can be expected to last at least 80 days, which markedly extends the observation period reported by others [25]–[31] thereby providing an opportunity for long-term experimentation with this model. The extent of colonization varied somewhat depending on the tested variable, but in general the fungal counts were in the range of 2–8×10^7^ CFU/g feces. The highest counts occurred several weeks after feeding of the fungus to C57BL/6 mice that were also treated with the immunosuppressant cyclophosphamide. As expected, the cyclophosphamide-treated mice did not respond by making antibodies to the presence of *C. albicans* colonization by day 43 (Fig. 1), but a low antibody titer was detectable by day 87 to the mannan extract (data not shown). Apparently, by this time the effect of the last cyclophosphamide treatment given on day 15 had waned, which is supported by the antibody response of the two survivors of this group to the Fba vaccine that was started on day 51 of the experiment (Fig. 1). The pilot experiment indicated that it was not essential to treat the animals with antibacterial and antifungal agents prior to a five day oral feeding with the fungus.

We anticipated that BALB/c mice should behave similarly. Additional control groups were added and to reduce the number of variables the immunosuppressant was not pursued in experiments with this mouse strain. BALB/c animals also readily developed a protracted GI-tract colonization with *C. albicans* following a five day period of oral feeding with the fungus. This colonization was antibiotic-dependent because BALB/c mice that did not receive antibiotics did not develop prolonged colonization following oral feeding with the fungus.

The health of all animals appeared normal by around day 11 of the experiments. In the pilot study, mice were prebled only 2–3 days prior to the start of the antibiotic-treated water for drinking. In that study, about 10% of the animals had died by day 5. In subsequent experiments, prebleeding five or more days before the start of the experiment resulted in almost 100% survival. We speculate that the loss of life in the pilot experiment could have been due to hypovolemia and probable toxic effects due to antibiotic-induced death of GI-tract microbial flora. The mice were initially reluctant to drink the antibiotic water, thus dehydration due to lack of drinking coupled with loss of blood during the prebleeding and the die-off of intestinal flora, may have been sufficient to cause the early deaths of a few of the animals. By day 11, however, the animals regained their weight and continued to thrive thereafter.

The sites of colonization within the GI tract were marked by the presence of the fungus in highest number in the caecum, followed by the colon. The stomach and small intestine were relatively light in fungal burden and the esophagus had barely detectable CFU's. This distribution within the GI tract is similar to the findings of others [27], even though our assessment was done 80–100 days into the colonization as compared to ∼20 days in the cited study. Although contradictory literature on *C. albicans* colonization sites in humans have been reported, several studies show similarities to results of our studies [35], which provides additional rationale for the use of the mouse model described in our work for studies on candidiasis pathogenesis.

### Horizontal transmission of the fungus

An unexpected finding occurred when antibiotic-treated mice that were not intentionally colonized by the oral route, but were housed in the same room as mice that received a five day oral feeding of *C. albicans,* also became colonized. The data strongly indicate that the transmission was due to the same strain as that used for intentional feedings. These findings ruled-out the possibility that another *Candida* species, such as *C. pintolopesia* that may be found in the GI-tract of some mice [36]–[39], was responsible for the spontaneous colonization of antibiotic treated animals. Importantly, antibiotic-treated mice not fed *C. albicans*, but kept in an isolated room, did not develop GI-tract colonization.

Our results allow us to speculate on the mechanism of horizontal transmission of *C. albicans* in the common room. First, we clearly show that the *Candida albicans* picked-up by the antibiotic-treated animals is the same strain as that used for intentional colonization. This result rules-out introduction of a strain of the fungus from an exogenous source, such as a strain that might be carried by the animal handlers. This is not surprising because all animal handlers routinely wear PPE, including gloves, face masks, bonnets, autoclaved gowns and shoe-booties as indicated in the Results section. It does not, however, necessarily rule out the possibility that investigators handling the fungal strains in the laboratory transmitted the strain when handling the experimental animals even though the investigators always wore appropriate PPE. Second, in the first experiment, which indicated the possibility of horizontal transmission, a common vessel was used to immobilize the animals to obtain weights and to collect stool specimens and gloves were not changed between cages. Third, in contrast to the above experiment, in the final experiment, which confirmed horizontal transmission, duplicate groups of antibiotic-treated mice not intentionally colonized were housed in an isolated room rather than in the common room containing intentionally and non-intentionally colonized mice. In this experiment, strict handling protocols dictated that personnel always handle animals in isolation before entering the common room, gloves were always changed between cages and separate sterile weighing vessels were used for each cage. These measures resulted again in horizontal transmission of the fungus in the common room, but colonization did not occur in the isolation room, which militates against a hypothesis that the fungal strain was introduced by investigators. However, the colonization was delayed in the common room as compared to the previous experiment. These findings have led us to conclude that antibiotic-treated mice are exquisitely sensitive to becoming colonized with *C. albicans.* The most likely mode of transmission is fomite contamination, and possibly aersols of bedding debris, that occurs while removing the cages from the ventilation environmental housing system and handling each of the animals during cage cleaning and while performing procedures to obtain animal weights and stool collections.

Since normal laboratory mice are not naturally colonized with *C. albicans*, and unusual measures are necessary to induce GI-tract colonization in the laboratory mouse [40], it was not surprising in our studies that non-antibiotic treated animals were highly resistant to *Candida* colonization even when intentionally fed the fungus (e.g., Fig. 2, group E). Furthermore, none of the non-antibiotic-treated animals developed colonization as a result of exposure to colonized mice in the common room (Fig. 2, groups F and I). The latter animals would be considered as normal laboratory mice, which have not been reported to being susceptible to horizontal transmission of the fungus. Our finding that antibiotic-treated mice are apparently exquisitely susceptible to horizontal transmission of *C. albicans* can be exploited in studies on host to host transmission of *C. albicans*. Indirect and direct human-to-human transmission of this and other *Candida* species is possible [41]–[43], but the mechanisms and prevention of transmission are not clear. Use of this animal model should allow experimentation to address both points.

### Development of serum antibodies in response to *Candida* colonization and vaccination

Mice may develop serum antibodies specific for cell surface antigens on the fungal cell wall in response to the colonization. Rules for predicting which of the animals would make a polyclonal antibody response were not obvious. For example, in the last experiment (Table 3 and Fig. 5), in two groups of mice intentionally colonized, 3/5 mice in group A developed serum antibodies, whereas in 5/5 mice in group G developed antibodies. These results show that mice may respond to the presence of *C. albicans* colonization by producing antibodies reactive with the fungal cell surface. Although not all colonized mice developed serum antibodies due to the presence of the fungus in their GI tract, a sufficient number of mice do develop antibodies which makes this model useful for determining whether animals making preformed antibodies against the fungus are capable of responding to a vaccine against candidiasis.

The Fba vaccine was chosen for these studies because we could readily distinguish between peptide vaccine-induced serum antibodies from antibodies that developed in response to the GI-tract colonization with the fungus. All mice that received the Fba vaccine responded by making specific antibodies against the peptide. The anti-Fba response occurred due to the vaccine rather than the colonization. Anti-Fba antibodies were not detected in sera of colonized animals prior to vaccination and at no time did colonized non-vaccinated animals make a detectable anti-Fba response. These results show that *Candida*-colonized mice, some of which with serum antibodies, are capable of responding to a vaccine against candidiasis. For purposes of human vaccination this is an important finding, because most humans are at least transiently colonized with the fungus in their GI tract and they have anti-*Candida* antibodies in their serum. In future experiments we will determine whether such animals respond to more complex synthetic glycopeptide conjugate vaccines already described [4], [44] and those that are in development in our lab, as well as improved delivery systems more suitable for human use. These future experiments will allow us not only to assess responsiveness of these mice to improved vaccine designs, but also whether vaccination affects GI-tract colonization with the fungus and how well vaccinated mice are protected against a live challenge with *C. albicans* and other *Candida* species.

In summary, adult mice can develop a chronic *C. albicans* GI-tract colonization of several months duration provided that their normal bacterial population is suppressed. Mice with a suppressed bacterial flora are exquisitely susceptible to acquiring *C. albicans* colonization by horizontal transfer of the fungus. Intentionally colonized animals and animals that become colonized by horizontal transmission may develop serum antibodies against *C. albicans* cell surface epitopes. Regardless of colonization and serum antibodies, however, all animals can develop a robust antibody response to a specific anti-*C. albicans* vaccine. We surmise that humans would be expected to respond to a vaccine against candidiasis even though they are colonized with *C. albicans* and have serum antibodies against the fungus prior to immunization.

## Materials and Methods

### Ethical Handling of Animals

All experiments involving mice were detailed and approved (protocol #120) by the Institutional Animal Care and Use Committee (IACUC) of the Research Institute for Children prior to performing any of the experiments. In accordance with IACUC policies, mice were housed exclusively in an AAALAC-certified facility. Details of experimental conditions and handling of animals are given in the appropriate Results section. All animals were sacrificed by CO_2_ asphyxiation when they became moribund as defined by a combination of ruffled fur, hunched back and dulled response to stimuli, such as finger probing. At the completion of all experiments, survivors were sacrificed by CO_2_ overdose in accordance with IACUC policy.

### Reagents

Penicillin G, streptomycin sulfate and gentamicin sulfate were obtained from Sigma Chem. Co., fluconazole was from LKT Labs, Inc. and all were supplied as a dry salt or powder. The preparations of each were penicillin G at 1.51 mg/mL, streptomycin sulfate at 2.02 mg/mL, fluconazole at 0.25 mg/mL and gentamicin at 0.2 mg/mL in autoclaved tap water and filter-sterilized (0.2 µm porosity filter) into oven-baked bottles. The various combinations given to the animals are indicated in Tables 1, 2, 3. Antibiotic-containing water was made fresh every seven days.

The fourteen amino acid residue, Fba, used to pulse dendritic cells in vitro and vaccinate the mice was obtained as a 95% pure synthetic product from GenScript as before [4]. A multiple antigenic peptide (MAP) on a lysine backbone to which multiple Fba moieties were covalently linked to the α- and ε-amino functional groups of lysine was obtained from GenScript. The MAP-Fba conjugate was used to coat wells for ELISA detection of antibody against the Fba as described below.

### Candida albicans

Strain SC3514 (ATCC MYA-2876) was the colonization strain, which was supplied to mice in their drinking water. The strain was retrieved 5–7 days before use in experiments from a water stock by aseptically placing a loopful into glucose (2%)-yeast extract (0.3%)-peptone (1%) (GYEP) broth, incubating under rotation (180 rpm) at 37°C for 18–24 h, transferring to fresh broth for another incubation, and continuing in this fashion for a minimum of three, but not more than 5 transfers before use. This method resulted in over 99% viable yeast forms that had a hydrophilic cell surface as previously described [45]. On the day of use, the cultures were examined microscopically to confirm yeast form growth and to check for bacterial contamination. The cultures were also plated onto GYEP agar plates and incubated for up to 4 days to confirm non-contamination. Yeast forms were harvested aseptically by centrifugation, washed 3 times in sterile tap water, counted by use of a hemocytometer and appropriately diluted into sterile drinking water containing penicillin and streptomycin to give 10^7^ SC3514 viable yeast/ml drinking water.

### Animals, blood collections and stool specimens

C57BL/6 and BALB/c female mice, 2–3 months, were obtained as 35 day old animals from Harlan Labs, Charles River Labs or the Jackson Laboratory. The basic strategy for GI-tract colonization of the mice with *C. albicans* was done as described by others [32], but with modifications as indicated in Tables 1 and 2. In a pilot experiment on C57BL/6 mice, animals in one group were rendered immunocompromised by giving intraperitoneal injections of a filter-sterilized solution of cyclophosphamide monohydrate (Fluka, Sigma-Aldrich) prepared in DPBS to provide a drug dose of 150 mg/kg on days 10, 12 and 14 of the experiment. Mice that received *C. albicans* in their drinking water were given water containing penicillin/streptomycin along with yeast forms of the fungus for days 5–9 (Tables 1, 2, 3). Groups of animals that received the Fba vaccine were given intraperitoneally a priming and first booster dose in the form of Fba-pulsed dendritic cells and a second booster subcutaneously as Fba emulsified in complete Freund adjuvant (CFA) (Imject Freund's Complete Adjuvant, Thermo Scientific) as previously described [4]. All non-vaccinated animals were given CFA alone as a control for an adjuvant effect. Prior to the experiments, a small sample (<200 µl) of blood was taken by cheek puncture from each animal, and again at various time points throughout the observation period to monitor for the presence of antibodies specific for mannan and Fba by ELISA as previously described [4], [9], [46]. Animal weights and stool specimens were obtained at specified times throughout the experiments. Stool specimens taken at various times were weighed, stored up to 2 weeks at −20°C, thawed and homogenized in sterile DPBS, appropriately diluted and plated for *C. albicans* colony forming units (CFU) on glucose (2%)-yeast extract (0.3%)-peptone (1%) containing 50 µg/ml chloramphenicol (Sigma). The storage of stools in the freezer was done for logistical purposes after we determined that similar CFU's were obtained from stools frozen for 24 h or up to two weeks. At specified times before and during antibiotic treatment, stool specimens were also processed to determine the presence of facultative anaerobic bacterial populations. Homogenates were plated onto trypticase soy agar containing 5% sheep blood (TSA II 5% SB, BBL) at 37°C. All plates were incubated for up to 4 days for evidence of bacterial growth. Fungal CFU's per gram fecal material were calculated, but bacterial growth was observed only for heavy growth, slight growth or no growth.

### Identification of *C. albicans* from the GI-tract

Several colonies from plated stool homogenates were randomly selected from various groups of colonized mice. Typical convex cream-colored colonies, 1–2 mm diameter *Candida*-like colonies developed from stool specimens and from all kidney homogenates by 24 h incubation of the plates at 37°C, but some specimens yielded also very small colonies (<1 mm diameter). However, all showed typical budding yeast cells with occasional pseudohyphal forms under microscopic exam. The colonial types and microscopic appearance were identical to that of strain SC5314 used for oral feedings. The various selected colonies and strain SC5314 were plated onto ChromAgar for comparison of pigment development. Multilocus sequence typing (MLST) was used for species and strain identifications/comparisons. The first analysis was done by use of primers from six selected housekeeping genes [47], [48] to produce PCR products from purified DNA from each isolate, the products of which were sequenced (Davis Sequencing). Subsequently two additional genes were added to the analysis as described [49]. The various PCR-amplified MLST products from each selected colony were compared by aligning the sequences using Sequencher (GeneCodes Corp., Ann Arbor, MI) and visual inspection of sequence identity.

### Detection of serum antibodies

ELISA was used to detect antibodies against cell surface mannan determinants and against the peptide, Fba, of *C. albicans.* For ELISA detection of antibodies primarily against mannan determinants, an extract of whole yeast form cells enriched for phosphomannoprotein complexes was obtained by treatment of the cells with 0.3 M 2-mercaptoethanol. We termed this the 2-ME extract and have characterized and used the 2-ME extract in ELISA assays numerous times [4], [9], [46], [50]–[52]. In brief, ELISA titers were obtained by coating polystyrene 96 flat bottom wells with the extract, washing the wells with Dulbecco's phosphate buffered saline (DPBS, Sigma) containing bovine serum albumin (Sigma), blocking by addition of 200 µl/well of DPBS containing 1% BSA, adding mouse serum samples starting at 1∶100 dilution in DPBS-BSA, incubating and washing with DPBS-BSA, adding secondary antibody (goat anti-mouse Ig-horse radish peroxidase, Sigma), washing with DPBS-BSA, adding 100 µl/well of substrate (*o*-phenylenediamine, Sigma), incubating for 10–20 min at 22–23°C, stopping the reaction by addition of acid and reading the OD of each well at 492 nm. Usually end-point titering was not done, but, rather the OD readings on various days were adjusted to results for a 1∶100 dilution of serum samples normalized to an internal control consisting of a known concentration of monoclonal antibody B6.1 specific for beta-linked oligomannosides of *C. albicans* [46].

To detect antibodies specific for the Fba peptide, wells were coated with the synthetic MAP-Fba conjugate (10 µg/mL) overnight 4 C, washed 2X PBS (0.01 M PB, 0.15 M NaCl, pH 7.4), blocked with 1% BSA in PBS, addition to wells of 100 µL of a 1∶100 of each antiserum (diluted in PBS), washed in PBS-0.05% Tween 20 3-5x, addition of secondary antibody (goat anti-mouse whole Ig-HRPO, Sigma), washings and addition of substrate, stop reagent and OD reading as above.
